# Changes in Low-Frequency Fluctuations in Patients with Antisocial Personality Disorder Revealed by Resting-State Functional MRI

**DOI:** 10.1371/journal.pone.0089790

**Published:** 2014-03-05

**Authors:** Huasheng Liu, Jian Liao, Weixiong Jiang, Wei Wang

**Affiliations:** 1 Department of Radiology, The Third Xiangya Hospital of Central South University, Changsha, Hunan, P.R.China; 2 Biomedical Engineering Laboratory, School of Geosciences and Info-Physics, Central South University, Changsha, Hunan, P.R.China; 3 Department of Information Science and Engineering, Hunan First Normal University, Changsha, Hunan, P.R.China; Hangzhou Normal University, China

## Abstract

Antisocial Personality Disorder (APD) is a personality disorder that is most commonly associated with the legal and criminal justice systems. The study of the brain in APD has important implications in legal contexts and in helping ensure social stability. However, the neural contribution to the high prevalence of APD is still unclear. In this study, we used resting-state functional magnetic resonance imaging (fMRI) to investigate the underlying neural mechanisms of APD. Thirty-two healthy individuals and thirty-five patients with APD were recruited. The amplitude of low-frequency fluctuations (ALFF) was analyzed for the whole brain of all subjects. Our results showed that APD patients had a significant reduction in the ALFF in the right orbitofrontal cortex, the left temporal pole, the right inferior temporal gyrus, and the left cerebellum posterior lobe compared to normal controls. We observed that the right orbitofrontal cortex had a negative correlation between ALFF values and MMPI psychopathic deviate scores. Alterations in ALFF in these specific brain regions suggest that APD patients may be associated with abnormal activities in the fronto-temporal network. We propose that our results may contribute in a clinical and forensic context to a better understanding of APD.

## Introduction

According to the Diagnostic and Statistical Manual Fourth Edition-Text Revision (DSM-IV-TR; American Psychiatric Association, 2000), the main symptoms of APD include deceitfulness, irresponsibility, disregard for rules, lack of remorse and impulsivity. Granted, repeat violent criminal behavior is a defining feature of APD. Based on a large-scale meta-analytic review of mental health in worldwide prison systems, Fazel and Danesh reported that 47% of male prisoners were diagnosed with APD, providing support for a close link between APD and criminal behavior [1]. In Finland, for example, men with APD are responsible for approximately 60 to 80% of the most serious violent offenses [2]. To date, the diagnosis of APD has been based entirely on antisocial behaviors (e.g., violation of social norms, irresponsibility, and criminality); whereas functional brain abnormalities are not well understood, understanding the pathophysiology of APD is clearly an international imperative. This close connection between APD and crime has urged researchers to employ brain imaging techniques to study individuals with APD. The hope is that a better understanding of the neurobiological basis of antisocial aggressive behavior can facilitate the development of a new generation of biosocial treatment and prevention programs to address a disorder with high societal costs, which has important implications in legal contexts and in helping to keep social stability.

Neuroimaging studies of individuals with APD have provided evidence of abnormalities in structure and function relative to control populations, with key impairments commonly found in the prefrontal and temporal cortices. For example, reduced prefrontal grey matter volume [3], reduced volume in temporal regions [4] and cortical thinning of the medial frontal lobe [5] had been found in antisocial adults. In addition, some studies examined the functional abnormalities in antisocial individuals using challenge tasks. Schneider and colleagues found an increase in activation in the dorsolateral prefrontal cortex and the amygdala during the acquisition phase of aversive conditioning in individuals with APD [6]. Using a working memory task, activation deficits in the left frontal gyrus and anterior cingulate cortex were found in violent APD patients compared with normal controls [7]. Using a visual/verbal working memory task, Raine et al. (2001) reported significant reduced activation in the right hemisphere, particularly in the temporal cortex, in the antisocial group compared to controls. As related constructs, psychopathy, which is generally considered a more severe syndrome of antisociality than APD, has much overlap in traits with APD [8]. Individuals with psychopathy have shown similar structural and functional prefrontal and temporal abnormalities to APD. A study by Kiehl and colleagues showed that criminal psychopaths failed to show the appropriate right anterior temporal activation when differentiating abstract from concrete words compared with healthy controls [9].

Recently, resting-state fMRI has been widely used in the investigation of brain functioning under normal and pathological conditions based on several advantages, including its high-resolution, no radiation use, and easy application [10]. Compared with traditional, task-related fMRI, the resting-state fMRI can be performed in any individual and is especially fit for individuals who are unable to cooperate with functional tasks [11]. To date, few resting-state fMRI studies have examined whether APD patients have an abnormal activities.

ALFF is considered a measure of resting-state local brain activity, which is similar to resting-state CBF and the glucose metabolic rate in PET studies. This hypothesis is supported by at least several pieces of evidence [12]. As a measure of the magnitude of the spontaneous BOLD signal, ALFF has been suggested to be associated with local neuronal activity, compared with degree of synchronization between activities in different regions measured by functional connectivity. ALFF has been widely used to study the “baseline” activity of a wide spectrum of psychological states and mental diseases, including aging, schizophrenia, and attention deficit hyperactivity disorder. Distributed differences in ALFF have been associated with different pathologies and mental states [13].

In our study, we utilized ALFF to investigate the alterations in resting state brain activities in APD patients compared to controls. These abnormalities may be a trait marker and could be helpful for the future diagnosis of APD patients. Based on previous studies, we hypothesized that an abnormal ALFF would exist in certain areas of the prefrontal-temporal network in APD patients contrast to controls.

## Materials and Methods

### Ethics Statement

All subjects in this study were of legal age to give consent (age 18 years old) to participate in the experiment, but they were under the legal age when they entered the School for Youth Offenders of Hunan Province. Written informed consent was obtained from all subjects after they were given a detailed description of the study; see Text S1 for details. This study was approved by the Ethical Committee of the Third Xiangya Hospital of Central South University and the School for Youth Offenders of Hunan Province.

### Participants

The subjects included in this study were the same as those included in our previous study [14], which included 32 APD individuals who met the APD criteria of the PDQ-4+ and DSM–IV and 35 controls without APD who were matched to the APD subjects in age and education. Volunteers were first tested using the Personality Diagnostic Questionaire-4+ (PDQ-4+), those had APD scores equal to or above 4 were further screened by the clinician-rated Personality Disorders Interview-IV (PDI-IV) [15], which is a semi-structured interview that yields a rating for each of the DSM-IV personality disorders, and those who met the APD criteria were diagnosed as APD. All of those tests were conducted by two senior psychiatrists. All subjects had been sentenced to reformatory education at this school for three years, and were native Chinese speakers. The participants had no access to alcohol and illicit drugs for at least six months prior to the brain scan. The subjects were strongly right-handed, as judged by the Lateral Dominance Test, and had normal or corrected-to-normal vision. No subjects from either group had a history of head trauma, chronic cardiac, hepatic, kidney or nervous system disease diagnosis, long-term drug dependency or metal implantation in vivo. After diagnosis by two senior psychiatrists, none of the subjects had a history or current diagnosis of serious mental disorders, e.g., depression, anxiety neurosis or schizophrenia. Individuals who did not cooperate during inspection were excluded. To further investigate their mental disorder situation and gauge psychopathy, the subjects completed handwritten versions of the MMPI-2 (Minnesota Multiphasic Personality Inventory) questionnaires. Specifically, MMPI-2's Pd (psychopathic deviate) subscale had yield promising results in the assessment of psychopathy [16].

All MR images were acquired in the Third Xiangya Hospital of Central South University. Each subject lay supinely, with the head snugly fixed by belt and foam pads to reduce the effects of head movement. In the experiments, the subjects were instructed simply to keep their eyes closed, relax, remain awake and perform no specific cognitive exercise. After each session, the subjects were asked whether they were awake in the previous session, and all of the subjects confirmed that they were awake. Magnetic resonance imaging scans were performed using a 1.5T Siemens Trio scanner using a gradient-echo EPI sequence. The imaging parameters are as follows: TR = 2000 ms, TE = 50 ms, FOV = 24 cm, FA = 90°, matrix = 64×64, slice thickness/gap = 5/1.2 mm, slices = 23. Each functional resting-state session lasted 5 min, and 150 volumes were obtained.

### Data Processing

In each subject, the first 5 volumes of the scanned data were discarded for magnetic saturation effects. The remaining 145 volumes were first preprocessed using SPM8. All subjects in this study had less than 1 mm translation along the x, y, or z-axis and 1° of rotation in each axis. After the head motion effect was removed, the volumes were normalized to the standard EPI template in the Montreal Neurological Institute (MNI) space. Smoothing and filtering were performed using a Gaussian filter at 8 mm full-width half-maximum kernel and a band-pass filter (0.01–0.08 Hz) respectively. Finally, the nuisance covariates, including head motion parameters, global mean signals, white matter signals and cerebrospinal fluid signals, were regressed from the images.

The ALFF analysis was then performed using the REST software. The ALFF calculation procedure was performed as follows: 1) Fast Fourier Transform (FFT) was used to convert all voxels from the time domain to the frequency domain; 2) the ALFF of every voxel was calculated by averaging the square root of the power spectrum across 0.01 Hz to 0.08 Hz; and 3) the resulting ALFF was converted into z-scores by subtracting the mean and dividing by the global standard deviation for standardization purposes [10]. A two-sample t-test was performed to explore the ALFF differences between APD patients and normal controls. The between-group statistical threshold was set at p<0.05 (FDR corrected) and cluster size>405 mm3 (15 voxels).

To explore whether the ALFF values of the brain regions that were significantly different between APD patients and controls could predict MMPI Pd subscale scores, we performed a correlation analysis. Partial correlations between the mean ALFF values and the MMPI Pd subscale scores were calculated with other subscale scores and demographics scores (age, IQ scores and years of education) and removed as confounding factors. The mean ALFF values were extracted from each cluster of voxels that showed the most significant differences between APD patients and normal controls.

## Results

As shown in table 1, there were no significant differences in demographics scores between two groups. On the ten MMPI subscales, APD patients were significantly higher than normal controls on the Hs (hypochondriasis), Pd (psychopathic deviate), Pa (paranoia), Pt (psychasthenia), Sc (schizophrenia) and Ma (mania) subscales, but they were significantly lower scores on the Hy (hysteria) subscale.

**Table 1:** Demographics and MMPI scores of APD patients and normal controls.

		APD patients (Mean±SD)	normal control (Mean±SD)	*p*
Demographics	Age	20.5±1.37	21.67±2.54	-
	Years of education	8.15±1.54	9.73±0.82	-
	IQ	106.66±12.90	106.84±16.6	-
MMPI	Hs: hypochondriasis	68.99±9.52	59.36±10.31	0.0018
	D: depression	50.64±11.05	55.34±8.99	-
	Hy: hysteria	51.73±8.73	57.57±10.78	0.044
	Pd: psychopathic deviate	72.92±9.11	58.18±10.01	3.52e-06
	Mf: masculinity–femininity	49.30±9.35	44.87±9.64	-
	Pa: paranoia	66.07±12.34	57.97±13.48	0.037
	Pt: psychasthenia	74.83±6.59	58.91±13.45	1.81e-06
	Sc: schizophrenia	75.64±8.40	57.08±12.40	1.51e-07
	Ma: mania	67.17±8.85	51.30±10.82	1.31e-06
	Si: social introversion	49.82±8.74	50.36±9.35	-

*p* with value ‘-indicates no significant difference between groups (p>0.05).

**Figure 1:**
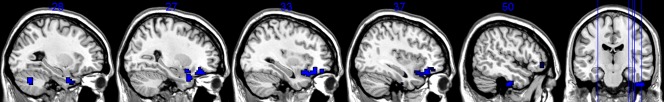
Statistical parametric map showing significant differences in the ALFF between APD patients and normal controls. The threshold for display was set to p< = 0.05 (FDR corrected), cluster size> = 405 mm^3^ (15 voxels). The regions with color represent decreased ALFF in APD patients.

As shown in table 2 and figure 1, when compared to normal controls, APD patients exhibited decreased ALFF mostly in the frontal cortex (including the right orbitofrontal cortex, which extended to the right ventrolateral prefrontal cortex, the insula and the anterior superior temporal cortex) and the temporal cortex (the middle and superior part of the left temporal pole; the anterior part of the right inferior temporal gyrus). In addition, the left cerebellum posterior lobe showed decreased ALFF. No region showed significant ALFF increases in the APD group.

**Table 2:** Brain regions exhibiting an altered ALFF between APD patients and normal controls.

Brain region	Cluster size	MNI coordinate	T value
		x y z	
APD patients<normal controls			
Right inferior temporal gyrus	31	51 −12 −39	−4.5212
Left temporal pole	28	−24 3 −30	−5.0663
Right orbitofrontal cortex	175	33 33 −21	−6.3501
Left cerebellum posterior lobe	36	−30 −63 −33	−5.2567

To explore whether the significantly different ALFF values in the brain regions of APD patients and controls could predict APD patients' psychopathic deviate scores, we correlated the MMPI Pd subscale scores and ALFF values of each cluster. We discovered that the right orbitofrontal cortex had a negative correlation between ALFF values and Pd scores (r = −0.5630, p = 0.0017).

## Discussion

The present fMRI study aimed to investigate the alterations in resting-state brain activities in APD patients; we found a significant fronto-temporal hypoactivity in APD patients when compared to normal controls.

The anterior temporal poles have rich connections to the amygdala, the basal forebrain, and prefrontal regions and are located near secondary auditory cortices and the termination of the ventral visual pathway. One prominent hypothesis of anterior temporal pole function, known as the Semantic Hub Account proposes that the anterior temporal poles serve as a hub and link sensory specific and semantic associations located throughout the brain [17].

In addition, new data showed that portions of the anterior temporal lobe play a critical role in representing and retrieving social knowledge. This knowledge includes memory about people, their names and biographies and more abstract forms of social memory such as memory for traits and social concepts [18]. In a study using a task that measures the meaning relatedness of social concepts, the results demonstrated that the superior anterior temporal cortex plays a key role in social cognition by providing abstract conceptual knowledge of social behaviors [19]. Another study confirmed the role of the anterior temporal poles in social semantic processing [20]. The same superior anterior temporal pole regions that represent abstract social concepts are recruited during the emotional judgment of social values and are stable across different contexts of moral sentiments [21]. Result Fronto Temporal Lobar Degeneration (FTLD) analyses revealed the right superior anterior temporal pole as associated with selective impairments of social concepts. These results corroborate the hypothesis that the right anterior temporal pole is necessary for representing conceptual social knowledge [22]. A perspective that may explain the involving of anterior temporal pole in social semantic processing state: ‘a general function of the temporal pole is to couple emotional responses to highly processed sensory stimuli. The mnemonic functions of this region allow for storage of perception–emotion linkages, forming the basis of personal semantic memory’ [23].

To date, studies have found structural and functional differences in the orbitofrontal cortex in antisocial individuals; these findings have been implicated as risk factors for antisocial personality disorder. A study by Adrian Raine et al. found that APD males showed an 8.7% reduction in orbitofrontal gray volume compared to male controls; reduced orbitofrontal volumes were significantly associated with increased APD symptoms and criminal offending in both males and females, indicating robustness of antisocial neuroanatomical relationships [24]. A meta-analysis of brain-imaging findings related to antisocial behavior that evaluated the relationship between prefrontal impairments and antisocial/violent/psychopathic behavior demonstrated that increased antisocial behavior was particularly associated with structural and functional reductions in the right orbitofrontal cortex [25]. Recently, our group used an exploratory data-driven classifier based on machine learning to investigate changes in functional connectivity in the brains of patients with APD using resting state functional magnetic resonance imaging, connectivities with the orbital part of right inferior frontal gyrus showed high discriminative power [14].

Another line of evidence linking orbitofrontal cortex dysfunction to antisocial personality disorder comes from numerous lesion studies, the parallels between the effects of orbitofrontal cortex lesions on social behavior and the symptoms of antisocial disorders are striking [26], this was confirmed by many studies. Tranel et al. showed that patients with unilateral lesions to the right orbitofrontal cortex were impaired in social conduct, decision-making, emotional processing, and personality [27]. Individuals with orbitofrontal cortex lesions are typically described as disinhibited, socially inappropriate, misinterpreting others' moods, impulsive, unconcerned with the consequences of their actions, irresponsible in everyday life, underestimating the seriousness of their condition (lack of insight), and showing a poor sense of initiative [26], [28].

The orbitofrontal cortex functions to any guide behavior that is based on the value of an expected reward or punishment, which thereby effects the decision made [29]. From this perspective, the results suggested that cocaine-induced changes in orbitofrontal cortex cause long-lasting impairments to the ability of orbitofrontal cortex to support outcome-guided decision-making and learning that results from comparing expected outcome to actual outcomes. During the Iowa Gambling Task (a decision-making task), cocaine abusers showed greater activation in the right orbitofrontal cortex, which may reflect that the potential for gain and reward trigger much stronger representations of reward-related thoughts in the orbitofrontal cortex in cocaine abusers [30]. Evidence also suggests that choosing high-risk over low-risk decisions was associated with increased activity orbitofrontal cortices, suggesting that the orbitofrontal cortex is preferentially involved in risky decision-making [31].

The Orbitofrontal cortex is also involved in emotion processes that guide social conduct. Incorrect orbitofrontal functioning determines the inability to generate or experience specific emotions that have a fundamental role in regulating individual and social behavior. According to the somatic marker hypothesis, individuals make judgments primarily in terms of their emotional quality. Interfering with the normal processing of somatic or emotional signals leads to pathological impairments in the decision-making process which seriously compromise the efficiency of everyday-life decisions [32]. By manipulating a simple gambling task, patients with orbitofrontal cortical lesions did not report regret or anticipate the negative consequences of their choices [33]. In an autobiographical memory paradigm comparing guilt shame and sadness, guilt episodes specifically recruited a region of right lateral orbitofrontal cortex, which was also highly correlated with trait guilt [34]. Anger has also previously been suggested to be specifically associated with the lateral orbitofrontal cortex [35]. The activation of a distinct system by angry expressions or expectations of another's anger results in the modulation of the current behavioral response in the individual engaging in the inappropriate behavior [36]. Furthermore, anger and aggression are often closely tied [37]. Damage to the orbitofrontal cortex impairs self-monitoring, leading to the conclusion that the orbitofrontal cortex is best conceptualized as an area important for self-monitoring processes that underlie the generation of emotions useful for guiding behavior [38]. A similar phenomenon was observed when the processing of transgressions of social norms involved systems previously found to respond to aversive emotional expressions (in particular angry expressions); namely, the lateral orbitofrontal cortex (Brodmann area 47) and the medial prefrontal cortex [36].

There are several reports of temporal and frontal lobe neuropathology co-occurring in aggression-prone individuals. Neuropathology occurring in both areas may increase the risk of angry aggression over that associated with neuropathology in either area alone [37]. The degeneration of the frontal and temporal lobes predisposes to a patient antisocial behavior, as supported by a relationship between frontal-temporal dysfunction and certain types of antisocial activities [39]. One study re-examined offenders convicted of lethal or near-lethal violence, the initially observed frontotemporal hypoactivity remained virtually unchanged at follow-up, suggesting that the frontotemporal brain hypoactivity is a trait, rather than a state in perpetrators of severe violent crimes [40]. Co-occurring temporal and frontal lobe neuropathology co-occurring has also been reported in psychopathy. Grey matter reductions were observed in the frontal (frontopolar, orbitofrontal, and insula) and temporal (anterior temporal cortices, superior temporal sulcus region) lobes of the psychopaths [41]. Müller J.L. et al. found significant gray matter reductions in the frontal and temporal brain regions in psychopaths compared with controls, especially a highly significant volume loss in the right superior temporal gyrus [42]. Further more, an impaired emotion–cognition interaction in psychopaths that correlated with changed prefrontal and temporal brain activation [43].

The anterior temporal pole is closely interconnected with cortical nuclei of the amygdala and the orbitofrontal cortex via the uncinate fasciculus. Ingrid R. Olson proposed that this pattern of connectivity underlies the function of the anterior temporal pole in encoding and storing emotionally tagged knowledge that is used to guide orbitofrontal-based decision processes [18]. An indirect evidence supporting for the orbitofrontal role in this propose came form orbitofrontal damage studies: adult patients with acquired sociopathy due to orbitofrontal damage have intact knowledge of social rules and norms, though they are unable (or unwilling) to use this knowledge to guide decision making [18], [32], [38], [44]. The functional and anatomic features of uncinate fasciculus also support this suggestion. The uncinate fasciculus is a long-range white matter association fiber tract that connects the orbitofrontal cortex to the anterior temporal lobes through a direct, bidirectional monosynaptic pathway. When using in vivo diffusion tensor magnetic resonance imaging tractography to analyse the microstructural integrity of the uncinate fasciculus in psychopaths. MC Craig reported a significant reduction in fractional anisotropy in the uncinate fasciculus of psychopaths. Within psychopaths, a correlation between measures of antisocial behavior and anatomical differences in the uncinate fasciculus was also found [45]. Frederick Sundram et al. found that the APD group had significantly reduced fractional anisotropy and significantly increased mean diffusivity compared with control subjects in the right uncinate fasciculus [8]. S. Sarkar et al. showed that adolescents with conduct disorder had significant differences in the ‘connectivity’ and maturation of uncinate fasciculus [46].

Michael Potegal proposed a similar model, but it focused on the temporal and frontal lobes' roles in the initiation and regulation of the top-down escalation of anger and aggression [37]. This model suggested that rudimentary appraisals of threat and provocation developed at the anterior temporal loc. At the same time, this modality-specific perceptual information is transmitted to the orbitofrontal cortex; these frontal areas then impose an inhibitory gating or modulation and focusing of activity initiated by the anterior temporal loci. In this model, the role of the orbitofrontal cortex emphasizes not only the integration and evaluation of information but also self monitoring and control.

Roland Zahn provided additional evidence for the temporal and frontal lobe co-occurring. The results demonstrated that social values emerge from the co-activation of stable abstract social conceptual representations in the superior anterior temporal lobe and context-dependent different feeling contexts encoded in the fronto-mesolimbic regions. Specifically, superior anterior temporal lobe activity increased with conceptual detail in all conditions; indignation/anger activated lateral orbitofrontal-insular cortices [21], [47].

Finally, our study found right orbitofrontal cortex showed negative correlation between ALFF values and Pd scores. The correlation between Pd scale and psychopathy appears restricted to traits and characteristics associated with the social deviance facet rather than the interpersonal/affective facet [48]. We used only the MMPI scores in this study. It is possible that the ALFF in other regions is relates to some specific psychopathy subscales, and the relationships between psychopathy scores and ALFF changes may be found. In our future studies, more specific psychopathy score will be included.

Similarly, anatomical difference in the uncinate fasciculus in psychopaths negatively correlated with the ‘antisocial behavior’ (factor 2) facet of psychopathy [8], [45]. However, de Oliveira-Souza R et al. had found the degree of structural abnormalities in the frontal and temporal cortices were significantly related to the interpersonal/affective dimension of psychopathy [41], and frontopolar and anterior temporal lobe grey matter volume loss were reproduced as a distinctive features of individuals with psychopathy compared to individuals with antisocial personality disorder, but no psychopathy [49]. Future work is required to get a consistent result.

## Conclusion

In summary, our study used ALFF to examine the alterations in the resting state between APD patients and normal controls. We found that APD subjects have altered resting-state brain activity in broadly distributed brain areas that are involved in the fronto-temporal network. Combining these results with those of other studies, we propose that our results may contribute in a clinical and forensic context to a better understanding of antisocial personality disorder.

## Supporting Information

Text S1 The Details of the Informed Consent Procedures.
(PDF)

## References

[1] Fazel S, Danesh J (2002) Serious mental disorder in 23000 prisoners: a systematic review of 62 surveys. Lancet 359: 545–550. 11867106 10.1016/S0140-6736(02)07740-1

[2] Eronen M, Hakola P, Tiihonen J (1996) Factors associated with homicide recidivism in a 13-year sample of homicide offenders in Finland. Psychiatric services 10.1176/ps.47.4.403 8689372

[3] Raine A, Lencz T, Bihrle S, LaCasse L, Colletti P (2000) Reduced prefrontal gray matter volume and reduced autonomic activity in antisocial personality disorder. Archives of general psychiatry 57: 119. 10665614 10.1001/archpsyc.57.2.119

[4] Narayan VM, Narr KL, Kumari V, Woods RP, Thompson PM, et al (2007) Regional cortical thinning in subjects with violent antisocial personality disorder or schizophrenia. The American journal of psychiatry 164: 1418. 17728428 10.1176/appi.ajp.2007.06101631 PMC3197838

[5] Barkataki I, Kumari V, Das M, Taylor P, Sharma T (2006) Volumetric structural brain abnormalities in men with schizophrenia or antisocial personality disorder. Behavioural Brain Research 169: 239–247. 16466814 10.1016/j.bbr.2006.01.009

[6] Schneider F, Habel U, Kessler C, Posse S, Grodd W, et al (2000) Functional imaging of conditioned aversive emotional responses in antisocial personality disorder. Neuropsychobiology 42: 192–201. 11096335 10.1159/000026693

[7] Kumari V, Das M, Hodgins S, Zachariah E, Barkataki I, et al (2005) Association between violent behaviour and impaired prepulse inhibition of the startle response in antisocial personality disorder and schizophrenia. Behavioural Brain Research 158: 159–166. 15680203 10.1016/j.bbr.2004.08.021

[8] Sundram F, Deeley Q, Sarkar S, Daly E, Latham R, et al (2012) White matter microstructural abnormalities in the frontal lobe of adults with antisocial personality disorder. Cortex 48: 216–229. 21777912 10.1016/j.cortex.2011.06.005

[9] Kiehl KA, Smith AM, Mendrek A, Forster BB, Hare RD, et al (2004) Temporal lobe abnormalities in semantic processing by criminal psychopaths as revealed by functional magnetic resonance imaging. Psychiatry Research: Neuroimaging 130: 27–42. 14972366 10.1016/S0925-4927(03)00106-9

[10] Wen X, Wu X, Liu J, Li K, Yao L (2013) Abnormal Baseline Brain Activity in Non-Depressed Parkinson's Disease and Depressed Parkinson's Disease: A Resting-State Functional Magnetic Resonance Imaging Study. PloS one 8: e63691. 23717467 10.1371/journal.pone.0063691 PMC3661727

[11] Shimony JS, Zhang D, Johnston JM, Fox MD, Roy A, et al (2009) Resting-state spontaneous fluctuations in brain activity: a new paradigm for presurgical planning using fMRI. Academic radiology 16: 578–583. 19345899 10.1016/j.acra.2009.02.001 PMC2818666

[12] Xuan Y, Meng C, Yang Y, Zhu C, Wang L, et al (2012) Resting-state brain activity in adult males who stutter. PloS one 7: e30570. 22276215 10.1371/journal.pone.0030570 PMC3262831

[13] Di X, Kim EH, Huang C-C, Tsai S-J, Lin C-P, et al (2013) The influence of the amplitude of low-frequency fluctuations on resting-state functional connectivity. Frontiers in human neuroscience 7. 10.3389/fnhum.2013.00118 PMC3613753 23565090

[14] Tang Y, Jiang W, Liao J, Wang W, Luo A (2013) Identifying Individuals with Antisocial Personality Disorder Using Resting-State fMRI. PloS one 8: e60652. 23593272 10.1371/journal.pone.0060652 PMC3625191

[15] Widiger TA, Mangine S, Corbitt EM, Ellis C, Thomas G (1995) Personality Disorder Interview–IV: A Semistructured Interview for the Assessment of Personality Disorders: Psychological Assessment Resources Odessa, FL.

[16] Lilienfeld SO, Fowler KA, Patrick C (2006) The self-report assessment of psychopathy. Handbook of psychopathy 107–132.

[17] Skipper LM, Ross LA, Olson IR (2011) Sensory and semantic category subdivisions within the anterior temporal lobes. Neuropsychologia 49: 3419–3429. 21889520 10.1016/j.neuropsychologia.2011.07.033 PMC3192293

[18] Olson IR, McCoy D, Klobusicky E, Ross LA (2013) Social cognition and the anterior temporal lobes: a review and theoretical framework. Social cognitive and affective neuroscience 8: 123–133. 23051902 10.1093/scan/nss119 PMC3575728

[19] Zahn R, Moll J, Krueger F, Huey ED, Garrido G, et al (2007) Social concepts are represented in the superior anterior temporal cortex. Proceedings of the National Academy of Sciences 104: 6430–6435. 10.1073/pnas.0607061104 PMC1851074 17404215

[20] Ross LA, Olson IR (2010) Social cognition and the anterior temporal lobes. Neuroimage 49: 3452–3462. 19931397 10.1016/j.neuroimage.2009.11.012 PMC2818559

[21] Zahn R, Moll J, Paiva M, Garrido G, Krueger F, et al (2009) The neural basis of human social values: evidence from functional MRI. Cerebral cortex 19: 276–283. 18502730 10.1093/cercor/bhn080 PMC2733324

[22] Zahn R, Moll J, Iyengar V, Huey ED, Tierney M, et al (2009) Social conceptual impairments in frontotemporal lobar degeneration with right anterior temporal hypometabolism. Brain 132: 604–616. 19153155 10.1093/brain/awn343 PMC2724922

[23] Olson IR, Plotzker A, Ezzyat Y (2007) The enigmatic temporal pole: a review of findings on social and emotional processing. Brain 130: 1718–1731. 17392317 10.1093/brain/awm052

[24] Raine A, Yang Y, Narr KL, Toga AW (2009) Sex differences in orbitofrontal gray as a partial explanation for sex differences in antisocial personality. Molecular psychiatry 16: 227–236. 20029391 10.1038/mp.2009.136 PMC3008752

[25] Yang Y, Raine A (2009) Prefrontal structural and functional brain imaging findings in antisocial, violent, and psychopathic individuals: a meta-analysis. Psychiatry research 174: 81. 19833485 10.1016/j.pscychresns.2009.03.012 PMC2784035

[26] Séguin JR (2004) Neurocognitive elements of antisocial behavior: Relevance of an orbitofrontal cortex account. Brain and Cognition 55: 185. 15134852 10.1016/S0278-2626(03)00273-2 PMC3283581

[27] Tranel D, Bechara A, Denburg NL (2002) Asymmetric functional roles of right and left ventromedial prefrontal cortices in social conduct, decision-making, and emotional processing. Cortex 38: 589–612. 12465670 10.1016/s0010-9452(08)70024-8

[28] Rolls ET, Hornak J, Wade D, McGrath J (1994) Emotion-related learning in patients with social and emotional changes associated with frontal lobe damage. Journal of Neurology, Neurosurgery & Psychiatry 57: 1518–1524. 10.1136/jnnp.57.12.1518 PMC1073235 7798983

[29] Lucantonio F, Stalnaker TA, Shaham Y, Niv Y, Schoenbaum G (2012) The impact of orbitofrontal dysfunction on cocaine addiction. Nature neuroscience 15: 358–366. 22267164 10.1038/nn.3014 PMC3701259

[30] Bolla K, Eldreth D, London E, Kiehl K, Mouratidis M, et al (2003) Orbitofrontal cortex dysfunction in abstinent cocaine abusers performing a decision-making task. Neuroimage 19: 1085. 12880834 10.1016/s1053-8119(03)00113-7 PMC2767245

[31] Cohen M, Heller A, Ranganath C (2005) Functional connectivity with anterior cingulate and orbitofrontal cortices during decision-making. Cognitive Brain Research 23: 61–70. 15795134 10.1016/j.cogbrainres.2005.01.010

[32] Bechara A, Damasio H, Damasio AR (2000) Emotion, decision making and the orbitofrontal cortex. Cerebral cortex 10: 295–307. 10731224 10.1093/cercor/10.3.295

[33] Camille N, Coricelli G, Sallet J, Pradat-Diehl P, Duhamel J-R, et al (2004) The involvement of the orbitofrontal cortex in the experience of regret. Science 304: 1167–1170. 15155951 10.1126/science.1094550

[34] Wagner U, N'Diaye K, Ethofer T, Vuilleumier P (2011) Guilt-specific processing in the prefrontal cortex. Cerebral cortex 21: 2461–2470. 21427167 10.1093/cercor/bhr016

[35] Goldstein RZ, Alia-Klein N, Leskovjan AC, Fowler JS, Wang G-J, et al (2005) Anger and depression in cocaine addiction: association with the orbitofrontal cortex. Psychiatry Research: Neuroimaging 138: 13–22. 15708297 10.1016/j.pscychresns.2004.10.002

[36] Berthoz S, Armony J, Blair R, Dolan R (2002) An fMRI study of intentional and unintentional (embarrassing) violations of social norms. Brain 125: 1696–1708. 12135962 10.1093/brain/awf190

[37] Potegal M (2012) Temporal and frontal lobe initiation and regulation of the top-down escalation of anger and aggression. Behavioural Brain Research 231: 386–395. 22085875 10.1016/j.bbr.2011.10.049

[38] Beer JS, John OP, Scabini D, Knight RT (2006) Orbitofrontal cortex and social behavior: integrating self-monitoring and emotion-cognition interactions. Journal of cognitive neuroscience 18: 871–879. 16839295 10.1162/jocn.2006.18.6.871

[39] Miller BL, Darby A, Benson D, Cummings J, Miller M (1997) Aggressive, socially disruptive and antisocial behaviour associated with fronto-temporal dementia. The British Journal of Psychiatry 170: 150–154. 9093504 10.1192/bjp.170.2.150

[40] Anckarsäter H, Piechnik S, Tullberg M, Ziegelitz D, Sörman M, et al (2007) Persistent regional frontotemporal hypoactivity in violent offenders at follow-up. Psychiatry Research: Neuroimaging 156: 87–90. 17689934 10.1016/j.pscychresns.2006.12.008

[41] de Oliveira-Souza R, Hare RD, Bramati IE, Garrido GJ, Azevedo Ignácio F, et al (2008) Psychopathy as a disorder of the moral brain: fronto-temporo-limbic grey matter reductions demonstrated by voxel-based morphometry. Neuroimage 40: 1202–1213. 18289882 10.1016/j.neuroimage.2007.12.054

[42] Müller JL, Gänßbauer S, Sommer M, Döhnel K, Weber T, et al (2008) Gray matter changes in right superior temporal gyrus in criminal psychopaths. Evidence from voxel-based morphometry. Psychiatry Research: Neuroimaging 163: 213–222. 18662867 10.1016/j.pscychresns.2007.08.010

[43] Müller JL, Sommer M, Döhnel K, Weber T, Schmidt-Wilcke T, et al (2008) Disturbed prefrontal and temporal brain function during emotion and cognition interaction in criminal psychopathy. Behavioral sciences & the law 26: 131–150. 18327826 10.1002/bsl.796

[44] Saver JL, Damasio AR (1991) Preserved access and processing of social knowledge in a patient with acquired sociopathy due to ventromedial frontal damage. Neuropsychologia 29: 1241–1249. 1791934 10.1016/0028-3932(91)90037-9

[45] Craig MC, Catani M, Deeley Q, Latham R, Daly E, et al (2009) Altered connections on the road to psychopathy. Molecular psychiatry 14: 946–953. 19506560 10.1038/mp.2009.40

[46] Sarkar S, Craig M, Catani M, Dell'Acqua F, Fahy T, et al (2012) Frontotemporal white-matter microstructural abnormalities in adolescents with conduct disorder: a diffusion tensor imaging study. Psychol Med 1–11. 10.1017/S003329171200116X 22617495

[47] Green S, Ralph MAL, Moll J, Stamatakis EA, Grafman J, et al (2010) Selective functional integration between anterior temporal and distinct fronto-mesolimbic regions during guilt and indignation. Neuroimage 52: 1720. 20493953 10.1016/j.neuroimage.2010.05.038 PMC2941398

[48] Sellbom M, Ben-Porath YS, Stafford KP (2007) A comparison of MMPI–2 measures of psychopathic deviance in a forensic setting. Psychological assessment 19: 430. 18085935 10.1037/1040-3590.19.4.430

[49] Gregory S, Simmons A, Kumari V, Howard M, Hodgins S, et al. (2012) The antisocial brain: psychopathy matters: a structural MRI investigation of antisocial male violent offenders. Archives of general psychiatry: archgenpsychiatry. 2012.2222 v2011. 10.1001/archgenpsychiatry.2012.222 22566562

